# Soil Metagenomics Reveals Effects of Continuous Sugarcane Cropping on the Structure and Functional Pathway of Rhizospheric Microbial Community

**DOI:** 10.3389/fmicb.2021.627569

**Published:** 2021-03-05

**Authors:** Ziqin Pang, Fei Dong, Qiang Liu, Wenxiong Lin, Chaohua Hu, Zhaonian Yuan

**Affiliations:** ^1^Key Laboratory of Sugarcane Biology and Genetic Breeding, Ministry of Agriculture, Fujian Agriculture and Forestry University, Fuzhou, China; ^2^College of Agricultural, Fujian Agriculture and Forestry University, Fuzhou, China; ^3^Province and Ministry Co-sponsored Collaborative Innovation Center of Sugar Industry, Nanning, China; ^4^College of Life Sciences, Fujian Agriculture and Forestry University, Fuzhou, China; ^5^Center for Genomics and Biotechnology, Fujian Agriculture and Forestry University, Fuzhou, China

**Keywords:** sugarcane, continuous cropping, rhizosphere soil, metagenome, community structure, bacterial and fungal communities, functional genes

## Abstract

The continuous cropping of plants can result in the disruption of the soil microbial community and caused significant declines in yields. However, there are few reports on the effects of continuous cropping of sugarcane on the microbial community structure and functional pathway. In the current study, we analyzed the structural and functional changes of microbial community structure in the rhizospheric soil of sugarcane in different continuous cropping years using Illumina Miseq high-throughput sequencing and metagenomics analysis. We collected rhizosphere soils from fields of no continuous cropping history (NCC), 10 years of continuous cropping (CC10), and 30 years of continuous cropping (CC30) periods in the Fujian province. The results demonstrated that continuous sugarcane cropping resulted in significant changes in the physicochemical properties of soil and the composition of soil bacterial and fungal communities. With the continuous cropping, the crop yield dramatically declined from NCC to CC30. Besides, the redundancy analysis (RDA) of the dominant bacterial and fungal phyla and soil physicochemical properties revealed that the structures of the bacterial and fungal communities were mainly driven by pH and TS. Analysis of potential functional pathways during the continuous cropping suggests that different KEGG pathways were enriched in different continuous cropping periods. The significant reduction of bacteria associated with rhizospheric soil nitrogen and sulfur cycling functions and enrichment of pathogenic bacteria may be responsible for the reduction of effective nitrogen and total sulfur content in rhizospheric soil of continuous sugarcane as well as the reduction of sugarcane yield and sugar content. Additionally, genes related to nitrogen and sulfur cycling were identified in our study, and the decreased abundance of nitrogen translocation genes and *AprAB* and *DsrAB* in the dissimilatory sulfate reduction pathway could be the cause of declined biomass. The findings of this study may provide a theoretical basis for uncovering the mechanism of obstacles in continuous sugarcane cropping and provide better guidance for sustainable development of the sugarcane.

## Introduction

Soil microorganisms are essential and play critical roles in soil organic matter decomposition, nutrient availability, and cycling, and in some instances, improve stress tolerance or suppress pathogens to regulate the soil-borne diseases (Van Der Heijden et al., 2008; Sun et al., 2015). This complex plant-associated microbial community, is also referred to as the second genome of the plant (Berendsen et al., 2012). Changes in microbial communities affect the absorption and transformation of soil nutrients *via* the root system of plants (Beckers et al., 2017). In recent years, the important role of soil microbiome in regulating agricultural production and plant diseases has been elucidated in a large number of studies. For instance, a substantial number of bacterial strains of *Bacillus* isolated from rhizospheric soil samples of the sugarcane plants have N-fixation function and biocontrol property against two sugarcane pathogens (Singh et al., 2020). In addition, several studies on *Arabidopsis thaliana* have shown that the diverse soil microbiomes applied to the roots of *Arabidopsis thaliana* were able to modulate plant growth and the leaf metabolome (Badri et al., 2013). Moreover, soil microbiomes have the potential to help *Arabidopsis thaliana* plants deal with drought stress under *in vivo* conditions (Zolla et al., 2013). Hence, the study on soil microorganisms has a great potential to provide essential new insights into the impact of microbial diversity on plant growth and soil ecosystem functioning.

Continuous cropping refers to a system in which the same or similar crop is cultivated in the same soil year after year (Shipton, 1977). There are three main factors associated with continuous cropping: imbalance of soil nutrients, autotoxicity of root exudates, and shifts in microbial community composition (Zhu et al., 2018). However, long-term continuous cropping usually leads to soil-borne plant pathogen accumulation and crop yield reduction, which has been described as a continuous cropping obstacle (Garbeva et al., 2004; Liu et al., 2018). Recently, increasing numbers of studies such as peanut (Li et al., 2014), sweet potato (Gao et al., 2019), cotton (Xi et al., 2019), and soybean (Tian et al., 2020) have reported that continuous cropping resulted in the disruption of soil microbial community and caused significant declines in yields. Whereas, continuous cropping is common with multiple agricultural systems due to the limited arable land and inappropriate farming strategies in China (Lei et al., 2020). For these reasons, more attention should be paid to exploring the association and underlying mechanism of continuous monocropping and soil microbial community.

Sugarcane is an important economic tropical crop widely cultivated all around the world, providing 80% of the world’s sugar production, and it is also a crucial source of biofuel to ethanol production (Singh et al., 2020). Continuous cropping obstacles are common with sugarcane. In practice, the yield is severely hindered due to the continuous monocropping and thus becomes a bottleneck that hinders the sustainable development of the national sugarcane industry. Previous studies have focused on the effects of application of organic and inorganic amendments such as zinc and lime at different concentrations and postharvest straw burning on chemical properties, microbial diversity, microbial biomass, and functional genes in sugarcane-cultivated soils (Souza et al., 2012; Rachid et al., 2013; Val-Moraes et al., 2016; Navarrete et al., 2017). However, there are few studies on continuous sugarcane cropping in association with soil microorganisms.

In the last decade, most of the previous studies used 16S rRNA gene library construction and denaturing gradient gel electrophoresis (DGGE) methods to descript the soil microorganisms which are time and cost consuming and can only measure a small amount of dominant soil microbial groups (Chen et al., 2012; Xi et al., 2019). Besides, studies on functional analyses of soil microbiome are still relatively scarce. Recently, the emergence of a variety of molecular tools and the rapid development of next-generation DNA sequencing (NGS) technology, such as the metagenomics analysis have provided unprecedented opportunities for us to advance knowledge of composition and function of soil microbial communities (Navarrete et al., 2015).

Therefore, in this study, we applied the Illumina Miseq high-throughput sequencing technology to analyze changes and the effects in microbial community structure in the rhizospheric soil of sugarcane varieties in different continuous cropping years. We believe that (a) soil properties and sugarcane agronomic characters are considerably affected by continuous cropping, leading to shifts in soil microbial structure and diversity and (b) these variations in soil microbial community composition would reflect shifts in soil microbial function. In addition, we hypothesized that (c) the changes caused by continuous cropping will differ in the diversity of bacterial and fungal communities and may reduce community diversity. The overall results of this study will provide us with useful information on the differences of the soil microbial communities in sugarcane grown under continuous cropping conditions.

## Materials and Methods

### Sample Collection and Site Description

The rhizosphere soil samples were collected from Songxi town, Nanping city, Fujian province, China (longitude 118° 72′ E, latitude 27° 43′ N), at an altitude of 207 m, where the average annual rainfall is between 1,650 mm and the average yearly temperature is 19°C. The soil type was sandy loam (3.87% clay, 22.80% silt, 73.33% sand). Tillage and field management practices were the same in each sampling area for different sugarcane succession years; the planting density is approximately 96,000 buds per hectare. Sugarcane was planted in the early spring of 2017, 2008, and 1988, respectively, marked as “NCC,” “CC10,” and “CC30.” Before planting sugarcane in 2017 and 2008, the tillage history was a crop rotation practice of rice and legume crops. The sugarcane cultivars were all Chinese species (*Saccharum sinense* Roxb.), planted in mid-March of each year and harvested from December of that year to February of the next year. Each sugarcane cultivation trial site consisted of three replications with a 1.4-m spacing between sugarcane rows and a plot area ranging from 30.6 to 50.4 m^2^. All plots were fertilized with the traditional local fertilizer application of 300 kg/hm^2^ of urea, 75 kg/hm^2^ of K_2_O, and 300 kg/hm^2^ of calcium superphosphate per season. Thirty and seventy percent of the total fertilizer application were applied at the seedling and elongation stages of sugarcane, respectively. Sugarcane agronomic traits were investigated at the sugarcane maturation stage on 28 December 2017, and on that day, five rhizosphere soil samples were collected from each plot using the S-sampling method and mixed as a biological replicate. The collected soil samples were immediately stored in sterile plastic bags, placed in iceboxes, and brought back to the laboratory immediately. Then, all the samples were sifted through a 2-mm mesh and were thoroughly homogenized to further be divided into two parts: one part was air-dried to analyze the soil physical and chemical characteristics, while the rest of the samples were stored at −80°C until DNA extraction and determination of ammonium nitrogen and nitrate nitrogen.

### Analysis of Soil Physicochemical Properties and Crop Yield

Soil suspension with water (1:2.5 WV^–1^) was prepared in order to estimate soil pH using a pH meter (PHS-3C, INESA Scientific Instrument Co., Ltd., Shanghai, China). Soil total nitrogen (TN) and total sulfur (TS) in the extracts were assessed by using the Elemental Analyzer (vario MAX cube, Elementar, Germany). Molybdenum Blue protocol was followed in order to measure available phosphorus (AP) by utilizing hydrochloric acid and ammonium fluoride (Watanabe and Olsen, 1965). Available nitrogen (AN) was measured using the alkaline hydrolyzable diffusion method (Jian-Guo et al., 2009). Besides, available potassium (AK) was extracted by ammonium acetate and measured by flame photometry (Pansu and Gautheyrou, 2007). Soil organic carbon content (SOC) was measured by redox titration with 0.8 mol/L K_2_Cr_2_O_7_. Soil NH_4_^+^-N and NO_3_^–^-N were extracted with 1 mol/L KCl solution and determined on SmartChem140 Automatic Chemical Analyzer using fresh soil samples.

To measure the stalk diameter and height of the plants, 30 sugarcane plants were randomly selected in each field and measured with a measuring tape and Vernier caliper. Extech Portable Sucrose Brix Refractometer (Mid-State Instruments, CA, United States) was used to determine sucrose content and calculated through using the formula: sucrose (%) = Brix (%) × 1.0825 - 7.703. Sugarcane production was estimated using the following equations (Lin et al., 2013).

(a) Single stalk weight (kg) = [stalk diameter (cm)]^2^ × [stalk height (cm) − 30] × 1(g/cm^3^) × 0.7854/1,000
(b) Production (kg/hm^2^) = single stalk weight (kg) × productive stem numbers (hm^2^)

### Soil DNA Extraction and Metagenomic Sequencing

A *PowerSoil DNA Isolation Kit* (MoBio, Carlsbad, United States) was used to extract genomic DNA from rhizosphere soil samples following the manufacturer’s instructions. *NanoDrop 2000* spectrophotometer (*Thermo Scientific*, *Waltham*, *MA*, United States) was used to estimate the concentration and purification of soil DNA, and DNA quality was checked by 1% agarose gel electrophoresis. PCR products were purified using the *AxyPrep DNA Gel Extraction* Kit (Axygen Biosciences, United States). Then, PCR amplification was carried out using *TransStart Fastpfu DNA Polymerase* (AP221-02, Transgen Biotech, Beijing, China) with a 20 μl reaction system. Amplification was performed with an initial denaturation at 95°C for 3 min, followed by 27 cycles at 95°C for 30 s, annealing at 55°C for 30 s, and extension at 72°C for 30 s. The final extension was conducted at 72°C for 10 min. Then the amplified DNA was quantified using *QuantiFluor*^TM^-*ST* (*Promega*, *Madison*, *WI*, United States). Paired-end libraries were prepared using *TruSeqTM DNA Sample Prep Kit* (*Illumina*, San Diego, CA, United States). Adapters containing the full complement of sequencing primer hybridization sites were ligated to the Blunt-end fragments. Sequencing was performed on Illumina *Miseq PE 300* high-throughput sequencer (Majorbio Corporation, Shanghai, China). Finally, raw metagenomics and datasets were deposited in the NCBI Sequence Read Archive (SRA) database with a BioProject ID: PRJNA674450. This SRA submission will be released on 02 January 2021 or upon publication, whichever is first.

### Metagenomic Assembly and Gene Annotation

First, low-quality metagenomic reads (length < 50bp or with a quality value < 20) were removed by Sickle version 1.33^1^ (Wu et al., 2016). Next, contigs and scaffolds were assembled individually for each sample *via* Multiple_Megahit with default parameters and a minimum contig size of 300 bp. The open reading frames (ORFs) from each sample were predicted using MetaGene^2^, and the predicted ORFs with a length ≥ 100 bp were translated to amino acid sequences. Then, all gene sequences with an identity ≥ 0.9 and a coverage ≥ 0.9 were clustered to construct the non-redundant gene catalogs by CD-HIT^3^. High-quality reads were aligned to the non-redundant gene catalogs to calculate gene abundance by using SOAPaligner^4^. Moreover, BLASTP (BLAST Version 2.2.28 +^5^) was employed for the taxonomic and functional annotations of each sample by comparing the non-redundant gene catalogs with the NR database (*e*-value ≤ 1*e*^–5^) and KEGG database^6^ (Ogata et al., 1999), respectively.

### Statistical Analysis

Analysis of similarities (ANOSIM) function was performed in R version 3.2.1 using the vegan Package based on Bray-Curtis distance (Tian et al., 2020). Beta diversity was calculated by weighted UniFrac distance and analyzed by principal coordinate analyses (PCoA) (Lozupone and Knight, 2005). The Kruskal-Wallis sum-rank test and linear discriminant analysis (LDA) were used to analyze LDA effect size to find statistical differences in KEGG pathway (Segata et al., 2011). The “redundancy analysis (RDA)” function of the vegan package in R was used to conduct the RDA of multiple correlation variations among environmental factors and community composition at the genus level, and the environmental factors were fitted with the ordination plots using the vegan package in R with 999 permutations. One-way analyses of variance (ANOVA) with Tukey’s HSD multiple range tests were performed for multiple comparisons and the Spearman’s correlation coefficients were used to test the correlation significance between the soil properties and the abundances of KEGG level2 pathway were all calculated using SPSS v20.0 (SPSS Inc., Chicago, IL, United States) and then visualized by using pheatmap package in R. Differentially regulated genera of bacteria and fungi as determined by DESeq2^7^ (Love et al., 2014) were clustered using the R package Mfuzz^8^ (Lokesh and Matthias, 2019), which can group differentially regulated microbial genera into different clusters based on similar temporal expression patterns.

## Results

### Analysis of the Illumina Sequencing Data

In this study, after the Illumina sequencing, a total of 412,877,804 raw reads was obtained from 9 libraries. After filtering, 406,763,020 clean reads were identified and the percentage of clean reads relative to the raw reads in each library was above 98.52% (Supplementary Table 1). After assembling, a total of 2,532,654 contigs were identified, which has 1,391,612,009 bases. The N50 statistics showed that more than 50% of contigs were longer than 554 bp. The maximum contig of all genes was 1,063,704 bp, while the minimum length was 300 bp (Supplementary Table 1). In addition, the non-redundant gene catalog of bacteria and fungi was constructed and obtained 7,008,114 and 9,479 catalog genes, respectively.

### The Physicochemical Properties of Soil and Crop Yield From Three Time-Series Field

**FIGURE 1:**
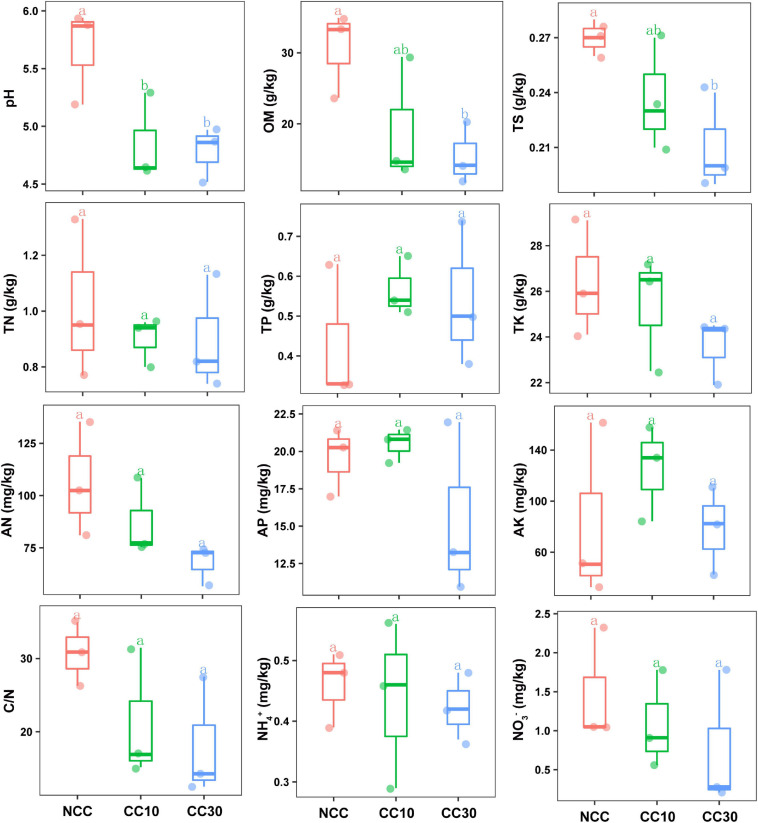
Effect of continuous sugarcane cropping on basic soil properties and nutrient contents. Boxes with the same lowercase letters indicate no significant difference between treatments based on the LSD test (*P* < 0.05). OM, organic matter; TN, total nitrogen; TS, total sulfur; TK, total potassium; TP, total phosphorus; AN, available nitrogen; AP, available phosphorus; AK, available potassium; NCC, no continuous cropping history; CC10, 10 years of continuous cropping; CC30, 30 years of continuous cropping.

**FIGURE 2:**
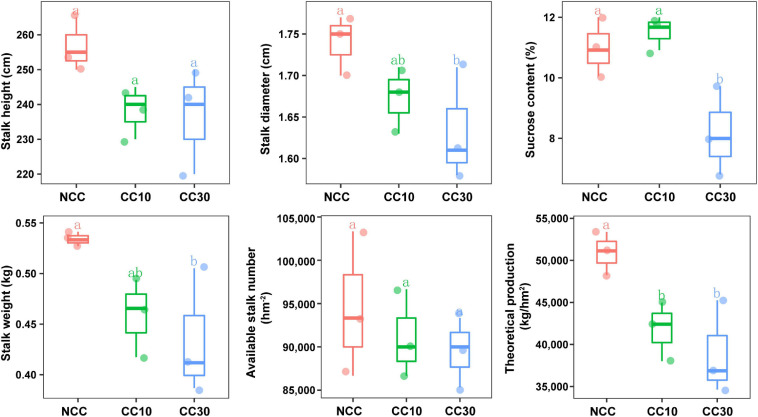
Effect of continuous sugarcane cropping on growth parameters, sucrose content, and yield of sugarcane. NCC, no continuous cropping history; CC10, 10 years of continuous cropping; CC30, 30 years of continuous cropping.

The results of a comparative analysis of soil physicochemical properties among the three time-series sampling sites, NCC, CC10, and CC30 (no continuous cropping and 10- and 30-year continuous cropping) are shown in Figure 1, respectively. With the increased years of sugarcane cultivation, soil pH, organic matter (OM), TN, TS, total potassium (TK), and AN contents significantly decreased, whereas the soil total phosphorus (TP) and AP slightly increased. Additionally, significant decreases in the sugarcane stalk height, weight, diameter, and production were observed after continuous cropping, while the available stalk number did not significantly differ for different years of continuous cropping with sugarcane (Figure 2).

### Bacterial Community Composition With Continuous Sugarcane Cultivation

**FIGURE 3:**
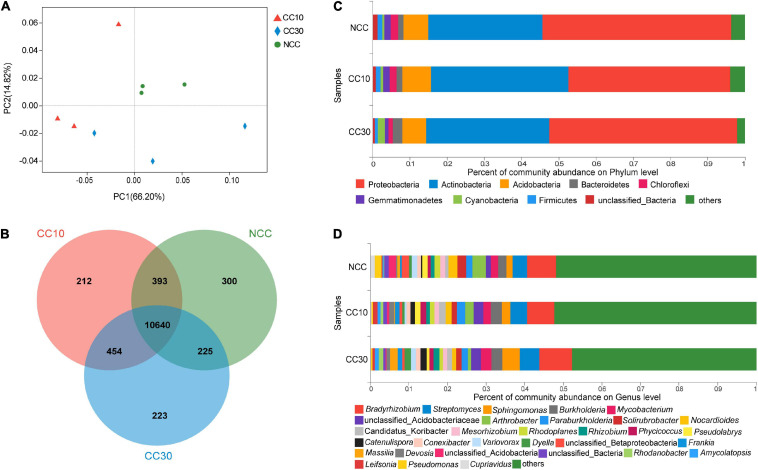
The microbiome compositions of bacterial taxa in three soil samples. **(A)** UniFrac-weighted PCoA plots of bacterial communities at phylum level in three samples. **(B)** Venn diagram of exclusive and shared bacterial species in three soil samples. **(C)** Bacterial community structure at the level of phylum. **(D)** The composition of bacterial community at the level of genus. NCC, no continuous cropping history; CC10, 10 years of continuous cropping; CC30, 30 years of continuous cropping; (phyla and genera with average relative abundance (RA) < 1% were merged and indicated as “Others”).

Principal coordinate analyses analysis of NCC, CC10, and CC30 rhizospheric soil bacteria was performed to compare the bacterial community among different samples (Figure 3A). The two main coordinates explained 81.02% of the microbial community changes among all the samples, of which PC1 explained 66.20% of the variation; PC2 explained 14.82% of the variation. Meanwhile, PCoA analysis shows that the samples were separated into three groups, samples of the CC10 group are much closer to the CC30 group due to their similar bacterial community structure, while the NCC group were distantly placed between three principal coordinates. This suggested that the structure of the bacterial community was affected after continuous cropping of sugarcane and indicated that the bacterial community structures in the CC10 and CC30 soil samples were distinctly different from the NCC soil samples. The ANOSIM results showed a similar result (Supplementary Table 2).

For the bacterial community, 82 phyla, 135 classes, 251 orders, 471 families, 1,895 genera, and 12,447 species were detected by metagenomic sequencing from three soil samples, of which 11,558, 11,699, and 11,542 species were detected in NCC, CC10, and CC30, respectively (Figure 3B). Among them, the dominant bacterial phyla in different years of continuous cropping of sugarcane were Proteobacteria, Actinobacteria, Acidobacteria, Bacteroidetes, Chloroflexi, Gemmatimonadetes, Cyanobacteria, and Firmicutes (relative abundance (RA) > 1%) (Figure 3C). The sum of these phyla accounted for more than 95% of the bacteriome, in which Proteobacteria (48.28%) and Acidobacteria (33.56%) accounted for the largest proportion. Moreover, the relative abundance of Proteobacteria, Chloroflexi, Gemmatimonadetes, and Firmicutes successively declined, while Bacteroidetes and Cyanobacteria significantly increased during the continuous cropping. Interestingly, the abundance of Actinobacteria and Acidobacteria shows the tendency increased at the beginning and declined lately.

To gain more insight into the variation on microbial abundance and composition during the continuous cropping, the microbial community was analyzed at the genus level. The analysis of variance was used to find the genera that were significantly enriched or reduced under different years of continuous cropping in sugarcane (Supplementary Table 3). The top 5 abundant bacterial genera in all groups were *Bradyrhizobium*, *Streptomyces*, *Sphingomonas*, *Burkholderia*, and *Mycobacterium*. Notably, with the increased year of continuous cropping, the proportion of *Streptomyces* and *Sphingomonas* and *Mycobacterium* was significantly higher in CC30 (5.13%, 4.49%, 0.94%) vs. NCC (3.82%, 1.66%, 0.55%), while *Arthrobacter* (from 3.52 to 0.80%), *Pseudomonas* (from 0.51 to 0.11%), and *Cupriavidus* (from 0.36 to 0.09%) showed the opposite result in significance (Figure 3D and Supplementary Table 3).

### Fungal Community Composition With Continuous Sugarcane Cultivation

**FIGURE 4:**
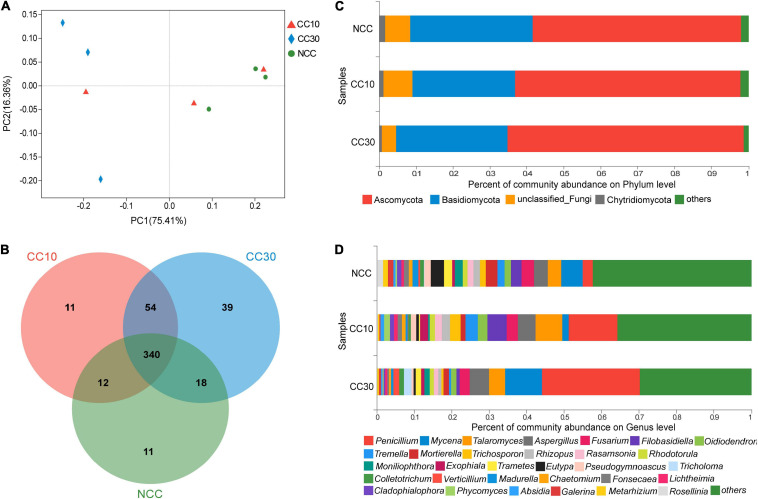
The microbiome compositions of fungal taxa in three soil samples. **(A)** UniFrac-weighted PCoA plots of fungal communities at phylum level in three samples. **(B)** Venn diagram of exclusive and shared fungal species in three soil samples. **(C)** Fungal community structure at the level of phylum. **(D)** The composition of fungal community at the level of genus. NCC, no continuous cropping history; CC10, 10 years of continuous cropping; CC30, 30 years of continuous cropping; (phyla and genera with average relative abundance (RA) < 1% were merged and indicated as “Others”).

The result of PCoA analysis of NCC, CC10, and CC30 rhizospheric soil fungal communities is shown in Figure 4A. The two main coordinates explained 91.77% of the variation, of which PC1 explained 75.41% of the variation and PC2 explained 16.36% of the variation. In addition, PCoA showed that the fungal composition of microbial communities differed between NCC and CC30. However, to some extent, the fungal compositions of NCC and CC10 have some similarities. The ANOSIM results showed a similar result in NCC vs. CC30 and NCC vs. CC10 (Supplementary Table 2).

For the fungal community, 10 phyla, 33 classes, 78 orders, 165 families, 282 genera, and 485 species were detected by metagenomic sequencing from three sugarcane soil samples. Three hundred eighty-one species were detected in NCC, 417 species in CC10, 451 species in CC30, and 340 were common to all three groups (Figure 4B). Among them, the dominant fungal phyla in different years of continuous cropping of sugarcane were Ascomycota, Basidiomycota, and Chytridiomycota (RA > 1%) (Figure 4C). The sum of these phyla accounted for more than 93% of the bacteriome, in which Proteobacteria and Acidobacteria accounted for the largest proportion (90.91%). Besides, the proportion of Ascomycota significantly increased (from 56.55 to 63.98%) with the increased years of continuous cropping of sugarcane, whereas Basidiomycota and Chytridiomycota decreased gradually from 33.09 to 30.23% and from 1.59 to 0.72%, respectively.

The analysis of variance was used to find the genera that were significantly enriched or reduced under different years of continuous cropping in sugarcane (Supplementary Table 4). At the genus level, *Penicillium*, *Mycena*, *Talaromyces*, *Aspergillus*, and *Fusarium* were the top 5 abundant fungal genera (RA > 2%). With the increased years of continuous cropping, the proportions of *Penicillium* and *Aspergillus* were on the rise in significance. Notably, the relative abundance of *Penicillium* was significantly higher after the continuous cropping and increased dramatically from 2.7 to 26.17%. However, *Eutypa* and *Pseudogymnoascus* gradually decreased during the continuous cropping from 3.52 to 0.49% and from 1.72 to 0.67%, respectively. Moreover, *Talaromyces*, *Oidiodendron*, and *Rhodotorula* were initially increasing and then declining in significance, while *Fusarium* showed a trend of first decline and then rise in significance (Figure 4D and Supplementary Table 4).

### Potential Functional Pathways in Sugarcane Rhizospheric Soil Microbes During the Continuous Cropping

Functional annotation was performed for 7,889,760 non-redundant genes based on the KEGG database and carbohydrate-active enzyme (CAZy) functional database to compare the relative abundances of potential functional genes during the continuous cropping stages. In total, 62,134,974 KEGG pathway-associated genes (Supplementary Table 5) and 2,428,520 CAZy genes (Supplementary Table 6) were detected from all metagenomes.

**FIGURE 5:**
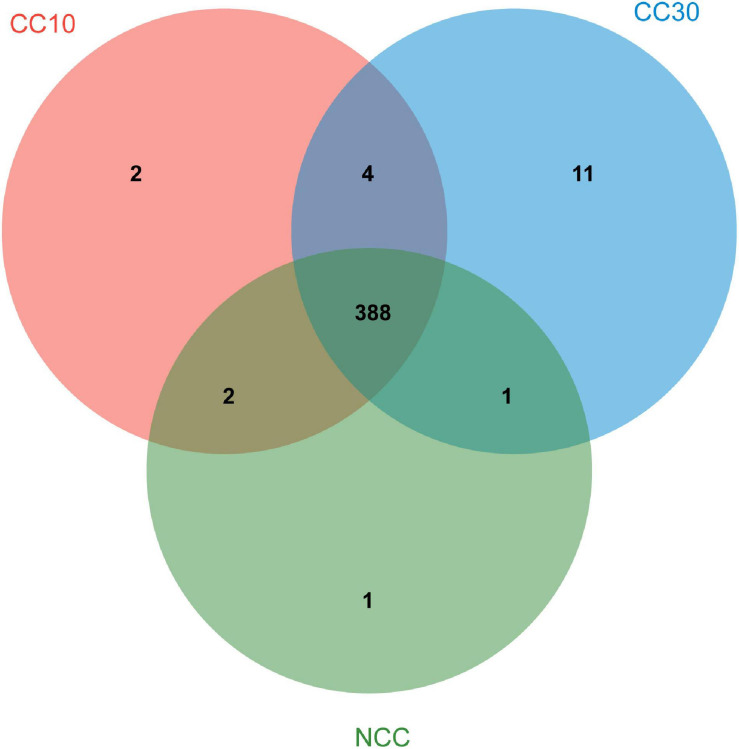
Venn diagram of exclusive and shared level 3 KEGG pathways in three soil samples.

As shown in Venn diagrams (Figure 5), a total 409 level 3 KEGG pathways were annotated from three sugarcane soil samples. Three hundred ninety-two pathways were detected in NCC, 396 pathways in CC10, 404 pathways in CC30, and 388 pathways were shared among three groups, while there were only one, two, and 11 unique pathways in NCC, CC10, and CC30, respectively. Among level 3 KEGG-annotated genes, the dominant categories were carbon metabolism, biosynthesis of amino acids, ABC transporters, quorum sensing, and two-component system, representing 4.93, 4.34, 3.44, 2.97, and 2.68%, respectively. Furthermore, among all categories, the relative abundances of genes related to the biosynthesis of amino acids, two-component system, purine metabolism, pyrimidine metabolism, glyoxylate and dicarboxylate metabolism, and oxidative phosphorylation increased during three continuous cropping stages. However, the relative abundances of genes associated with carbon fixation pathways in prokaryotes, methane metabolism, and nitrogen metabolism decreased with time.

**FIGURE 6:**
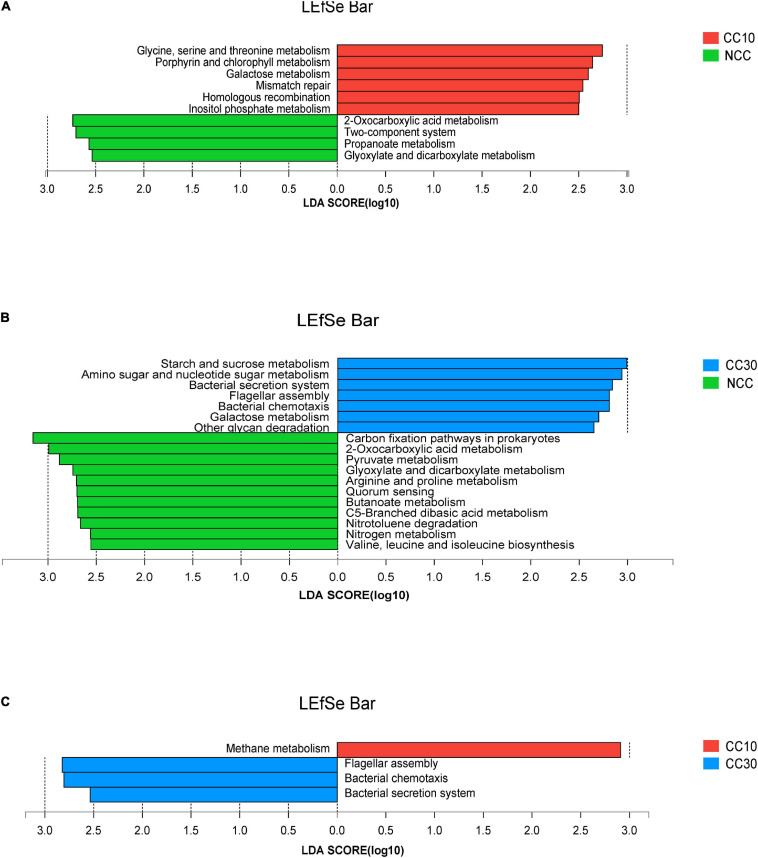
LEfSe analysis of level 3 KEGG pathway with significant differences based on during the continuous sugarcane cropping. **(A)** NCC vs. CC10; **(B)** NCC vs. CC30; **(C)** CC10 vs. CC30. Lineages with LDA values higher than 2.5 are displayed.

The LEfSe analysis was conducted on all level 3 KEGG pathways to determine pathways at the level 3 KEGG gene annotation with significant differences in abundance during the continuous cropping. Persistent differences in KEGG pathway patterns between the different continuous cropping years were revealed (Figure 6). When comparing NCC and CC10, NCC was primarily associated with 2-oxocarboxylic acid metabolism, two-component system, propanoate metabolism, and glyoxylate and dicarboxylate metabolism, while CC10 was primarily associated with glycine, serine, and threonine metabolism, porphyrin and chlorophyll metabolism, galactose metabolism, mismatch repair, homologous recombination, and inositol phosphate metabolism. Additionally, when comparing NCC and CC30, NCC was mainly associated with carbon fixation pathways in prokaryotes, 2-oxocarboxylic acid metabolism, pyruvate metabolism, glyoxylate and dicarboxylate metabolism, arginine and proline metabolism, quorum sensing, butanoate metabolism, C5-branched dibasic acid metabolism, nitrotoluene degradation, nitrogen metabolism, and valine, leucine, and isoleucine biosynthesis, while CC30 was mainly associated with starch and sucrose metabolism, amino sugar and nucleotide sugar metabolism, bacterial secretion system, flagellar assembly, bacterial chemotaxis, galactose metabolism, and other glycan degradation. Moreover, CC10 was dominantly associated with methane metabolism, while CC30 was dominantly associated with flagellar assembly, bacterial chemotaxis, and bacterial secretion system when comparing CC10 with CC30.

In addition, the annotated CAZy were assigned to six classes and the proportion of glycoside hydrolases (40.98%) was the highest, followed by glycosyl transferases (27.39%), carbohydrate esterases (17.17%), carbohydrate-binding modules (8.12%), auxiliary activities (4.67%), and polysaccharide lyases (1.67%). Furthermore, the abundance of all CAZy categories was increased over time (Supplementary Table 6).

### Effect of Environmental Factors on Bacterial and Fungal Community Composition and Functional Pathways

We firstly used variance inflation factors (VIF) and Spearman’s correlation analysis to perform environmental factor screening and evaluate the interaction between environmental factors. The high autocorrelation environmental factors were removed, and the environmental factors with less interaction (VIF < 10) were selected to do further study (Supplementary Table 7). Spearman’s correlation analysis demonstrates whether the interaction between environmental factors is significant (Supplementary Table 8). RDA and Spearman’s correlation analysis were then conducted to define the environmental factors influencing the microbial structure and functional pathway in the soil samples, respectively.

**FIGURE 7:**
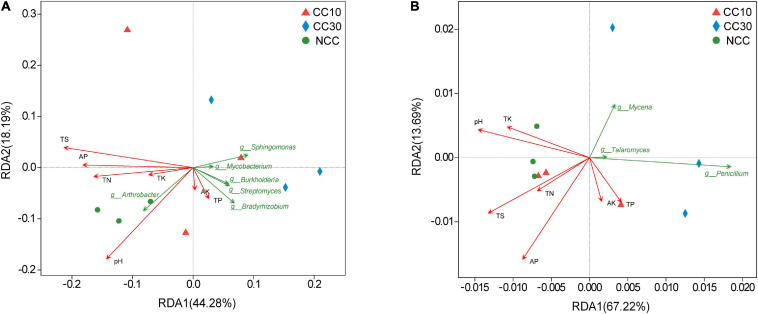
Redundancy analysis plots of the correlation between microbial community and environmental factors during continuous cropping. **(A)** Bacterial community; **(B)** fungal community.

The RDA results about the bacterial and fungal community are visualized in Figure 7. It suggested that the first two RDA components (RDA1 and RDA2) explain 44.28 and 18.19% of the total variance in bacterial community, respectively, and 67.22 and 13.69% (RDA1 and RDA2), in fungal community. Besides, we calculated the *r*^2^ and *P* values to investigate the significances of the effects of soil environmental factors on microbial community composition. Among these soil environmental factors, significant influences of pH (*r*^2^ = 0.857, *P* value = 0.005), TS (*r*^2^ = 0.773, *P* value = 0.02), and AP (*r*^2^ = 0.543, *P* value = 0.091) were observed in bacterial community structure, as well as the significant influences of TS (*r*^2^ = 0.614, *P* value = 0.047) and AP (*r*^2^ = 0.822, *P* value = 0.022) on fungal community composition, indicating that bacterial and fungal community composition was strongly affected by pH, TS, and AP during the continuous cropping.

**FIGURE 8:**
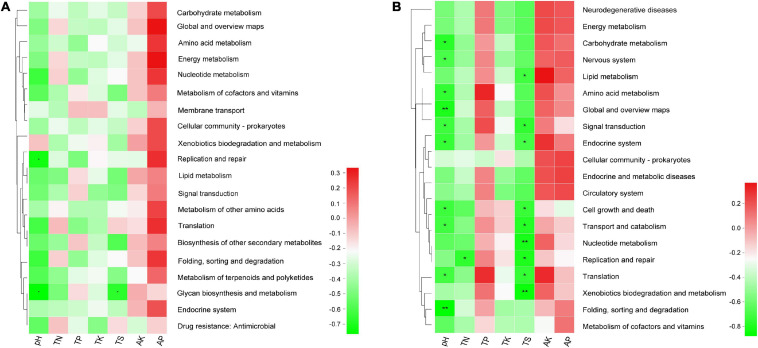
The heatmap of the correlation between level 2 KEGG pathway traits and physicochemical characteristics of rhizospheric soil **(A)** in the bacterial community and **(B)** in the fungal community. This heatmap was created according to the result of Spearman’s correlation analysis. Positive relationships are represented in red, while negative relationships are represented in green. The significant correlations are presented as asterisks (*P < 0.05; **P < 0.01).

Moreover, the results of Spearman’s correlation analysis in Figure 8 revealed the relationships between KEGG level 2 pathway traits and environmental factors. In bacterial community, the replication and repair pathway abundance showed a negative correlation with pH (*r* = −0.733, *P* = 0.025), glycan biosynthesis and metabolism pathway abundance also showed a negative relationship with pH and TS (*r* = −0.767, *P* = 0.016 and *r* = −0.669, *P* = 0.049), respectively (Supplementary Table 9.1). In the fungal community, several pathways have significant negative association with pH, for example, folding, sorting, and degradation (*r* = −0.867, *P* = 0.002) and global and overview maps (*r* = −0.8, *P* = 0.01) with the *P* value < 0.01. In addition, pathways like nucleotide metabolism (*r* = −0.812, *P* = 0.008) and xenobiotics biodegradation and metabolism (*r* = −0.845, *P* = 0.004) have significant negative association with TS (Supplementary Table 9.2).

### Nitrogen and Sulfur Cycling in Microbial Communities During Sugarcane Continuous Cropping

**FIGURE 9:**
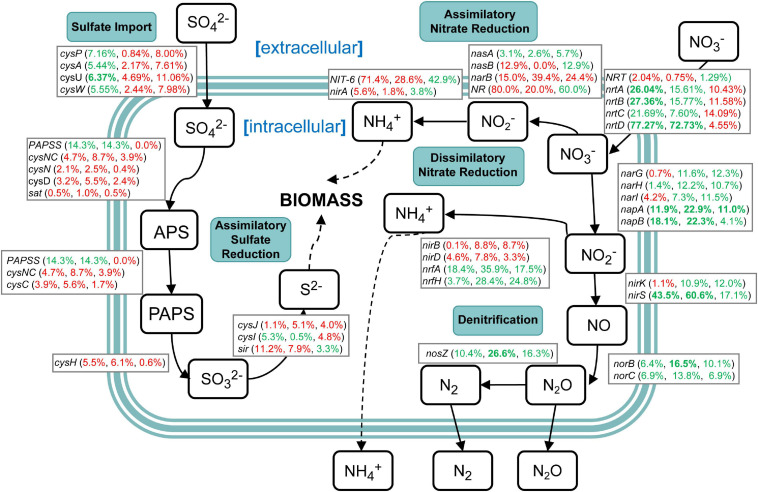
A diagram representing selected nitrogen and sulfur pathways and the difference in abundance of the underlying genes between NCC, CC10, and CC30 metagenomes. Percentages are ordered like this: CC10 vs. NCC, CC30 vs. CC, and CC30 vs. CC10. Percentages given in red or green represent an increase or decrease in the abundance of the corresponding genes, respectively. Significant results are bolded (*P* < 0.05).

Genes related to nitrogen and sulfur cycling were identified based on the results of metagenomic KEGG annotations (Supplementary Table 10). The results showed that various enzymes encoded by corresponding functional genes took part in the nitrogen and sulfur metabolism in sugarcane rhizospheric soil. However, there were a variety of changes in the abundance of genes related to nitrogen and sulfur cycling (Figure 9). In the sulfur cycle, sulfate import genes *cysFUWA* were on average 6.13% more abundant in the NCC group rather than in the CC10 group, while CC30 showed an average 2.54% increased abundance compared with NCC. Following step is assimilatory sulfate reduction, which may convert sulfate into new biomass. A majority of these genes showed a slight increase during continuous sugarcane cropping including *cysND*, *sat*, *cysH*, *cysJ*, and *sir*, while *PAPSS* and *cysI* decreased. In the nitrogen cycle, except for *nasA*, the rest of the assimilatory nitrate reductase genes *nasB*, *narB*, *NR*, *NIT-6*, and *nirA* were more abundant in the CC30 and CC10 groups. Moreover, genes specific to dissimilatory nitrate reduction including *narH*, *napAB*, and *nrfAH* decreased during continuous sugarcane cropping while *nirBD* increased as continuous cropping years increased. Besides, *narG* and *narI* increased from NCC to CC10 then decreased during CC10 to CC30. Genes specific to denitrification like *nirK/S*, *norBC*, and *nosZ* generally decreased in abundance during NCC to CC30. Nevertheless, genes related to the transfer of extracellular nitrate into cells including *nrtABCD* were more abundant in the NCC group rather than in the CC10 and CC30 groups, especially *nrtD* gene manifested an over 70% decline abundance compared with NCC.

## Discussion

Continuous cropping obstacles are common phenomena in various plants, including medicinal plants (Tang et al., 2015; Alami et al., 2020), fruits (Hui-Yan and Yan-Song, 2009; Li and Liu, 2019), and especially in many crops (Xi et al., 2019; Tian et al., 2020), which have adverse impacts on plant health resulting in great economic losses. However, continuous cropping obstacles are caused by complex factors, and the mechanisms remain unclear. Recently, an increasing number of studies have begun to focus on the roles of soil microbial communities in continuous cropping practices (Xiong et al., 2015b; Gao et al., 2019; Chen et al., 2020). Sugarcane, one of the important tropical crops in China, has been planted in monoculture in coastal areas of southern China due to the limited arable land area, which has resulted in soil sickness and significant losses to crop yields could be caused by changes in the microbial composition and soil characteristics (Miura et al., 2016; Kelly et al., 2018). Thus, unveiling soil microbial community and function variations under continuous cropping system is extremely helpful in understanding the association between reduced crop yields and long-term monoculture.

Previous studies have shown that continuous cropping affected the physicochemical properties of rhizosphere soil, thus reducing the nutrient absorption capacity of the plant’s root (Alami et al., 2020). In this study, the decrease of pH, OM, TN, TS, and TK was observed in the continuous cropping of sugarcane fields, probably because of the application of organic fertilizer and the gradual decrease in nitrogen, sulfur, and potassium utilization capacity of the soil microbial community (Chao et al., 2014). Additionally, the TP and AP showed an increasing trend with the increase of cultivation years, which may result from low Pi utilization, the misuse of inorganic fertilizers, and soil P sorption capacity increasing due to the low soil pH (Hou et al., 2020; Zhang W. et al., 2020). The above changes in physicochemical properties of soils might limit the sugarcane growth and contribute to the decrease of sugarcane production after continuous cropping.

Microbial community composition and diversity are associated with soil quality and plant health (Berendsen et al., 2012). A large number of studies have shown that there are significant differences in the microbial biomass and composition of bacterial and fungal communities at different stages of monoculture (Gao et al., 2019; Alami et al., 2020; Chen et al., 2020). Interestingly, the diversity of bacterial communities increased from NCC to CC10, and then decreased from CC10 to CC30 in this study. We found Proteobacteria and Actinobacteria were the dominant bacterial phyla in the bacterial communities at all stages of monoculture, which is consistent with numerous previous studies. The phylum Proteobacteria are dominant members of the rhizosphere microbial communities and enriched in the rhizosphere soil of plenty of plants (Peiffer et al., 2013; Philippot et al., 2013). Besides, the phylum Actinobacteria belongs to Gram-positive bacteria, and it could produce antibiotics to inhibit plant pathogens in soil and playing a crucial role in the decomposition of organic matter (Zhang H. et al., 2017). Previous studies have shown that the increase in Proteobacteria and Actinobacteria is associated with long periods of intense production which may be consistent with the increased abundance caused by continuous crop in our study (Zhongmin et al., 2018). Our results also have shown the relative abundances of Actinobacteria, Bacteroidetes, and Cyanobacteria increased while that of Proteobacteria, Chloroflexi, Gemmatimonadetes, and Firmicutes decreased over time. In previous studies, the main reason for the increase of Actinobacteria abundance in rhizosphere soil was the decrease of soil pH caused by organic acid secreted by roots (Haichar et al., 2014). Besides, Cyanobacteria were negatively correlated with soil N fractions in our study, which was inconsistent with the positive correlation between soil Cyanobacteria abundance and soil N content reported in previous studies (Xu et al., 2017; Ren et al., 2020). The phylum Proteobacteria is one of the most diverse and fastest metabolism in bacteria which plays an important role in maintaining soil ecological stability by soil nitrogen supply (Ren et al., 2020). It reported that Chloroflexi abundance showed a positive correlation with soil pH (Khodadad et al., 2011) and could be decreased through regulating soil available N, available P, and available K, which is consistent with the results of our study. Moreover, previous studies have shown that the abundance of Gemmatimonadetes was particularly affected by environmental factors and soil types and positively related to soil moisture content (Ren et al., 2020). This may mean that the changes after continuous cropping in this study may be related to the decreased water-holding capacity, which needs to be verified by further experiments. The phylum Firmicutes had a positive correlation with the stem length, stem diameter, and fiber yield of continuous ramie. Firmicutes was the dominant phylum in continuous ramie soils (Zhu et al., 2018), but it accounted for only 0.81–1.26% in this study. These results suggested that each continuous cropping system had different mechanisms, especially in the composition of the bacterial community.

At the genus level, *Bradyrhizobium* is symbiotic nitrogen-fixing bacteria which perhaps evolved from photosynthetic free-living bacteria by the acquisition of symbiotic functions (Molouba et al., 1999). *Streptomyces* is the major Actinomycetes genus which can help to produce several antibiotics that are useful in medical practices as well as an important role in organic matter decomposition conducive to crop production (Qin et al., 2011). *Sphingomonas* belongs to Gram-negative bacteria that are widely distributed in nature. Some *Sphingomonas* strains showed the ability of nitrogen fixation and denitrification in the nitrogen cycle and play a crucial role in reducing the toxic substances in soil (Hu et al., 2007; Yang et al., 2016). *Burkholderia* is a kind of Gram-negative bacteria that belongs to the Proteobacteria. Most of *Burkholderia* are progrowth bacteria that can promote the growth and development of plants through nitrogen fixation, nodulation, and phosphorus solubilization (Gomes et al., 2003). Our study showed that the abundance of *Bradyrhizobium*, *Streptomyces*, *Sphingomonas*, and *Mycobacterium* was relatively high in CC30 and that *Arthrobacter*, *Pseudomonas*, and *Cupriavidus* were more abundant in NCC. The *Arthrobacter* are Gram-positive, non-acid-resistant, aerobic, chemoheterotrophic bacteria that are the dominant bacteria in water and soil. It is also the plant growth-promoting bacteria (PGPR) capable of degrading nicotine and resisting insect attack (Brown, 1974). In addition, a strain HS-G_8_ of *Arthrobacter* with biological nitrogen fixation ability was isolated from the soil in Okinawa Prefecture, Japan (Jiang et al., 2004). *Pseudomonas* can strengthen the cell wall of plant roots against pathogens by inducing changes in plant cell wall structure (Benhamou, 1996). *Cupriavidus* is a Gram-negative bacterium of the order β-Amastigotes, which is widely found in the environment. It can tolerate and adsorb heavy metal ions and increase the pH of the soil microenvironment (Zhaohui et al., 2014). Therefore, these genera may play a more important role in continuous sugarcane cropping.

Fungi play a key role in decomposition, and the composition of soil fungi community is affected by management and soil nutrient status (Sui et al., 2019). Among the fungi identified in our study, the diversity of fungal communities increased from NCC to CC30 and a similar result was also reported in continuous cropping of vanilla and peanut (Xiong et al., 2015b; Chen et al., 2020). The dominant phyla of fungi in continuous cropping of sugarcane were Ascomycota, Basidiomycota, and Chytridiomycota, which was consistent with previous studies in continuous cropping of sweet potato and *Panax notoginseng* (Tan Y. et al., 2017; Gao et al., 2019). Interestingly, we observed the opposite situation for the increased proportion of Ascomycota during continuous cropping, which was decreased in other studies (Feng et al., 2019; Gao et al., 2019). The relative abundance of Basidiomycota and Chytridiomycota decreases gradually. At the genus level, the relative abundance of *Penicillium* and *Aspergillus* increased during continuous cropping, indicating the increase of harmful microorganisms. Members of the genus *Talaromyces* are well known for their secondary metabolites, with some having antimicrobial activities (Zhai et al., 2016). *Fusarium* is a soil-borne pathogen which can persist in the soil for a long time without any host. Numerous *Fusarium* species have been reported as the dominant pathogens of many crops (Naeem et al., 2019). *Eutypa* belongs to the Ascomycetes; the secondary metabolites from *Eutypa* have antibacterial and immunosuppressive bioactivities (Tan J.J. et al., 2017). Thus, the significant decrease of *Eutypa* may lead to a negative impact on disease resistance during continuous cropping.

**FIGURE 10:**
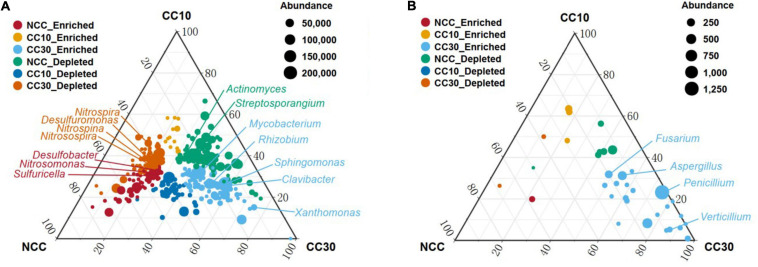
Ternary plot depicting soil microorganisms with significant differences in abundance in three sugarcane soil samples. **(A)** Ternary plot of genus level in bacterial community with significant differences in abundance. **(B)** Ternary plot of genus-level in fungal community with significant differences in abundance. Vertex represents NCC, CC10, and CC30, respectively; each point corresponds to a species, and the size of the point represents the relative abundance of each species. Axes show reads accounted for by each species in each group of soil samples (NCC, CC10, and CC30), as a percentage of total (sum) reads observed for a given species across all three groups. Arrows indicate the corresponding axis directions for each point. “Enriched” representative compared with other two groups of abundance has significantly increased; “Depleted” representative compared with the other two groups have significantly lower abundance.

To analyze the overall distribution of microorganisms during the continuous sugarcane cropping, ternary plots were used for visualization in domain and phylum level, respectively (Supplementary Figure 1). In domain level, we observed an enrichment of Archaea in NCC and a subset of Eukaryota and viruses were highly enriched in CC30. In phylum level, Gemmatimonadetes and Rokubacteria were enriched in NCC, while Bacteroidetes showed enrichment in CC10 and CC30. Interestingly, a subset of Cyanobacteria was highly enriched in CC30 compared with both NCC and CC10. Together, these results suggest that microbial composition may change with continuous cropping of sugarcane and that there are specific microbial species at different periods. All these specific microbial species may have complex functions and mechanisms that need to be intensively investigated in the future. We further used analysis of variance to identify genera that were significantly enriched or reduced during different years of continuous cropping which can explain how continuous sugarcane cultivation affects the rhizosphere soil microbial communities (Figure 10 and Supplementary Figure 2). For the bacterial community, CC30 showed a significant increase in 187 genera and a significant decrease in 164 genera compared with NCC, with no significant change in the remaining 1,544 genera. The abundance of bacterial genera associated with nitrogen and sulfur cycling were significantly decreased in CC10 and CC30 compared with NCC, including *Nitrosospira*, *Nitrosospina*, and *Nitrospira* associated with nitrogen cycling (Singh et al., 2020), and 12 *Desulfobacter* including *Desulfuromonas* associated with sulfur cycling. In contrast, the abundance of phytopathogenic bacteria *Clavibacter*, which can cause systemic plant diseases such as wilt, foliage, and bacterial canker disease (Nandi et al., 2018); *Mycobacterium*, which comprises more than 177 species which is pathogenic to both animals and humans (Sarbashis et al., 2018); *Rhizobium* and *Sphingomonas*, associated with nitrogen fixing (Molouba et al., 1999; Hu et al., 2007); and *Xanthomonas*, a genus of pathogenic bacteria hazardous to agricultural production (Bonas and Boch, 2010), increased significantly. Meanwhile, *Streptosporangium*, an Actinomycete known to produce antimicrobial compounds was also significantly increased (Hardoim et al., 2015). For the fungal community, CC30 showed a significant increase in 38 genera and a significant decrease in three genera compared with NCC, with no significant changes in the remaining 241 genera. *Penicillium* is a toxin-producing genus that can cause fruit, vegetable, and meat rots, as well as citrus penicillium (Chen et al., 2020); *Aspergillus*, which can produce toxic secondary metabolites (Dagenais and Keller, 2009); *Fusarium*, which can cause plant rot, stem rot, flower rot, and spike rot (Naeem et al., 2019); and *Verticillium*, which can cause plant *Verticillium* wilt (Jian-Guo et al., 2009), were significantly enriched in CC30; and the first three genera increased with the years of continuous cropping in sugarcane. This suggests that long-term continuous cropping of sugarcane may lead to a decrease in the function of nitrogen and sulfur cycling in the rhizosphere soil of sugarcane, as well as the enrichment of pathogenic bacteria that are pathogenic to plants or animals. The above changes in microbial abundance may be closely related to the decrease in effective nitrogen and total sulfur content of sugarcane inter-root soil, sugarcane yield reduction, and sugar content decrease under long-term continuous cropping.

Principal coordinate analyses is a non-constrained data dimensionality reduction analysis method that can be analyzed at different levels to observe the differences between individuals or groups (Lozupone and Knight, 2005). ANOSIM analysis is a non-parametric test used to test whether the differences between groups (two or more groups) are significantly greater than the differences within groups to determine whether the groups are significant (Tian et al., 2020). The result of the UniFrac-weighted PCoA and ANOSIM in this study demonstrated that the continuous cropping of sugarcane had strong effects upon the soil microbial community structure. This result was consistent with previous studies that soil microbial community structure significantly changed with the years of continuous cropping of black pepper, cucumber, and *Coptis chinensis* (Zhou and Wu, 2012; Xiong et al., 2015a; Alami et al., 2020). Therefore, we further supported the hypothesis that soil microbial communities could be affected by continuous cropping and may contribute to the poor sugarcane growth in continuous crop cultivation.

Different metabolic pathways could lead to different physiological consequences. As shown in our study, carbon metabolism, biosynthesis of amino acids, ATP-binding cassette (ABC) transporters, quorum sensing, and two-component system were enriched in soil microorganisms regardless of the continuous cropping years. According to the KEGG database, carbon metabolism is the most basic aspect of life, contains carbon utilization pathways of glycolysis, pentose phosphate pathway, and citrate cycle, and six carbon fixation pathways, as well as some pathways of methane metabolism. Biosynthesis of amino acids is a modular architecture that includes the biosynthesis pathways of 20 amino acids. The ABC transporters form one of the largest known protein families and are widespread in bacteria, archaea, and eukaryotes. Quorum sensing (QS) is a regulatory system that allows bacteria to share information about cell density and adjust gene expression accordingly. Two-component signal transduction systems enable bacteria to sense, respond, and adapt to changes in their environment or their intracellular state (Ogata et al., 1999). Moreover, NCC was primarily associated with glyoxylate and dicarboxylate metabolism, carbon fixation pathways in prokaryotes, nitrotoluene degradation, and nitrogen metabolism; these pathways mainly related to energy metabolism. Especially, carbon fixation is an important pathway for autotrophs living in various environments, and the biological process of nitrogen metabolism is a complex interaction of many microorganisms involved in the flow of energy through oxidation and reduction. However, CC10 and CC30 were associated with bacterial secretion system, flagellar assembly, and bacterial chemotaxis. Bacterial secretion system is related to a wide range of protein secretion including biogenesis of organelles, such as flagella, nutrient acquisition, virulence, and efflux of drugs and other toxins. The bacterial chemotaxis is the process by which cells sense chemical gradients in their environment and then move toward more favorable conditions. This interaction causes a change in behavior, such as in direction or speed of rotation of flagella. Some researches indicated that flagellar assembly is related to type III virulence secretion systems and can be used for the induction of maximum fluid secretion (Cui et al., 2013). Virulence factors are molecules that play very important roles in enhancing the pathogen’s capability in causing diseases. Metabolism pathways, flagellar assembly, and chemotaxis, relating to cellular motility, may be of importance for virulence (Berkelmann et al., 2020). In addition, increases in abundance of marker genes for flagellar assembly, chemotaxis, and types VI and IV secretion systems could indicate that an increment of motility and interaction in soil microbial communities accompanies sugarcane continuous cropping.

Carbohydrate-active enzyme genes have the functions of degradation, modification, and generation of glycosidic bonds. CAZy genes were classified into six classes, including auxiliary activities (AA), carbohydrate-binding modules (CBM), carbohydrate esterases (CE), glycoside hydrolases (GH), glycosyl transferases (GT), and polysaccharide lyase (PL) (Lombard et al., 2014). Further research on CAZy genes is of great significance for revealing the metabolic mechanism of microbial carbohydrates. The composition and abundance of CAZy genes during continuous cropping were characterized in our study. All these classes were found in three soil samples. GH is the class with the highest abundance which can hydrolyze the glycosidic bond through the addition of a water molecule and catalyze the hydrolysis of glycosidic linkages to generate smaller polysaccharides/monosaccharides (Lombard et al., 2014). Nevertheless, the number of CAZy genes in CC30 was higher than in NCC and CC10. This result is unexpected and requires further study.

Continuous cropping obstacles refer to crop yield reductions that occur in monocultures, which are often caused by the degradation of soil ecosystems such as declining pH, increased pathogenic bacteria, and decreased numbers of beneficial microorganisms. According to some reported studies, soil physicochemical properties have important roles in controlling microbial community structure (Lauber et al., 2008; Val-Moraes et al., 2016). In addition, many bacterial communities are highly correlated with specific soil factors and can be used as indicators of soil condition (Kuramae et al., 2015). Investigating the correlation between microbial community diversity and soil environmental factors can help us better understand the mechanism of continuous cropping obstacles. In our study, the RDA results suggest that many soil properties may have affected microbial community structures. We found that the structures of the bacterial and fungal communities were mainly driven by pH and TS. The Spearman’s correlation analysis shows that pH has no significant influence on the TS (Supplementary Table 8). Previous research suggests that soil pH has strong effects on the soil microbial community composition and diversity (Rousk et al., 2010). For instance, the abundance of fungi in soil is highly influenced by soil pH and fungal growth was increased 30-fold in acidic soils (pH = 4.5) (Rousk et al., 2009). The main reason for continuous soybean cropping obstacle is the decrease in soil pH (Tian et al., 2020). Besides, in the bacterial community, we found that the *Arthrobacter* abundance showed a positive correlation with pH and that *Bradyrhizobium*, *Sphingomonas*, *Streptomyces*, and *Burkholderia* showed a negative relationship with pH and TS. In the fungal community, the relative abundance of the *Mycena*, *Penicillium*, *Talaromyces*, and *Aspergillus* were all negatively correlated with pH and TS. The heatmap of the correlation between the top 20 genus and physicochemical characteristics showed that most genera were significantly correlated with TN and TS in the bacterial community and pH and TS in the fungal community, respectively. Previous studies also revealed the correlations between pH and fungal abundance. For instance, TN has been shown to affect soil microbial community structure and diversity (Rousk et al., 2009). TS in the soil leads to an increase in microorganisms contained in the soil (Klotz et al., 2011). Thus, the TN and TS content may be related to the nitrogen and sulfur cycling changes in microbial communities during continuous sugarcane cropping. Moreover, the relationships between KEGG level 2 pathway traits and environmental factors revealed that the abundance of replication and repair, glycan biosynthesis, and metabolism pathway are negatively correlated with pH in bacterial community, which was in coherence with a recent study showing that glycan and amino acid metabolism were pH sensitive (Ricky et al., 2018). In the fungal community, most of the top 20 KEGG level 2 pathways were significantly negatively correlated with pH and TS which is consistent with the relationship between fungal composition and environmental factors.

Soil microbial biomass is an essential indicator of soil quality and reflects the process of nutrient transfer and the energy cycle (Powlson et al., 1987). In previous studies, soil enzyme activities and soil microbial biomass always have a strong positive correlation (Fall et al., 2016; Zhang Y. et al., 2020). The N cycle consists of complex interplay pathways including assimilatory nitrate reduction, dissimilatory nitrate reduction, denitrification, nitrogen fixation, nitrification, and anammox. There are a variety of genes encoding enzymes that catalyze the important transformation reactions of various oxidation states ranging from + 5 in nitrate to −3 in ammonia (Ren et al., 2017). N is a critical limiting factor for continuous sugarcane cropping yield, while excessive application of N fertilizer will lead to problems like soil acidification and high cost (Yang et al., 2019). About nitrogen metabolism, we propose two possibilities to explain the relationship between the N cycle and biomass in our study. The first is that despite the increased abundance (no significant difference) of most genes in assimilatory nitrate reduction, the dramatic decrease (significant difference) of extracellular nitrogen translocation was a gradual restriction for substrates that participate in the intracellular N cycle which eventually decreased sugarcane biomass. Moreover, decreases of *NRT* and *nrtABCD* may result in a limitation of nitrogen, and induce chlorosis and inhibit chloroplast protein translation in the previous study (Plumley and Schmidt, 1989). The second is that the decrease of denitrification leads to produce more ammonia within the cell. Then the accumulated nitrites and ammonia might act as toxins, resulting in the decreased biomass like a previous study (Safdar et al., 2017). Sulfur is an essential element for life and occurs in various oxidation states ranging from + 6 in sulfate to −2 in sulfide (H_2_S). Sulfate reduction can occur in both an energy-consuming assimilatory pathway and an energy-producing dissimilatory pathway. The transformation of sulfur in the environment is critically dependent upon microbial activities (Klotz et al., 2011). In our study, the abundance of most genes (except *PAPSS* and *cysI*) in the energy-consuming assimilatory sulfate reduction pathway were increased, meanwhile, the abundance of genes (*AprAB* and *DsrAB*) in the energy-producing dissimilatory pathway were decreased during continuous cropping. This may be one of the contributors to decreased biomass. Thus, changes in relative abundances of functional genes in nitrogen and sulfur cycling indicated that shifts within soil microbes and functional pathways may have impacts on soil microbial biomass reflected in the growth of the plant eventually.

## Conclusion

In this study, we have shown that continuous cropping in sugarcane cultivation led to significant declines in soil pH, OM, TN, TS, TK, and AN contents. The metagenomic sequencing and analysis confirmed our proposed hypothesis that continuous cropping reduces the diversity of bacterial and fungal communities and that soil microbial community structure and function were significantly affected by the continuous sugarcane cropping system. The dominant bacterial phyla were Proteobacteria, Actinobacteria, and Acidobacteria, and the dominant fungal phyla were Ascomycota, Basidiomycota, and Chytridiomycota. The reduction in diversity and abundance of beneficial soil microbes like *Rhizobium* and *Sphingomonas*, and an increase in harmful soil microbes like *Mycobacterium*, *Fusarium*, and *Verticillium*, could be the main reason for sugarcane poor growth and crop diseases. Variations in microbial community diversity and functional pathways were mainly caused by differences in pH, TN, and TS. In nitrogen metabolism, the dramatic decrease of nitrogen translocation from extracellular to intracellular and the accumulated nitrites and ammonia by the decreased denitrification could result in the decreased biomass. In sulfur cycling, the decreased abundance of *AprAB* and *DsrAB* in the energy-producing dissimilatory sulfate reduction pathway together with the increased abundance of most genes (except *PAPSS* and *cysI*) in the energy-consuming assimilatory sulfate reduction pathway might be the contributors to the decreased biomass. Significant decreases in the abundance of bacterial genera associated with nitrogen and sulfur cycling, such as *Nitrosomonas*, *Desulfobacter*, *Sulfuricella*, and related functional genes may have contributed to the reduction of the energy cycle. All these changes might finally result in sugarcane crop yield and quality reduction during continuous cropping. This study provides a theoretical basis on the mechanism underlying obstacles in continuous cropping systems of sugarcane. However, the specific cause of continuous cropping obstacles was still uncertain. Further consideration must be given to the application of microbial methods such as exploring sustainable agricultural measures and special microbial fertilizers.

## Data Availability Statement

The datasets presented in this study can be found in online repositories. The names of the repository/repositories and accession number(s) can be found below: https://www.ncbi.nlm.nih.gov/, PRJNA674450&lt.

## Author Contributions

All authors contributed to the intellectual input and provided assistance to this study and manuscript preparation. ZY and ZP designed the research and conducted the experiments. FD analyzed the data and wrote the manuscript. QL, WL, and CH reviewed the manuscript. ZY supervised the work and approved the manuscript for publication.

## Conflict of Interest

The authors declare that the research was conducted in the absence of any commercial or financial relationships that could be construed as a potential conflict of interest.

## Footnotes

1 https://github.com/najoshi/sickle

2 http://metagene.cb.k.u-tokyo.ac.jp/

3 http://www.bioinformatics.org/cd-hit/

4 http://soap.genomics.org.cn/

5 http://blast.ncbi.nlm.nih.gov/Blast.cgi

6 http://www.genome.jp/kegg/

7 https://bioconductor.org/packages/release/bioc/html/DESeq2.html

8 https://bioconductor.org/packages/release/bioc/html/Mfuzz.html

## Supplementary Material

Supplementary Figure 1 Ternary plot depicting RAs of soil microorganisms in three sugarcane soil samples. **(A)** Ternary plot of domain level from all species among NCC, CC10, and CC30 soil samples. **(B)** Ternary plot of phylum level from top 300 most abundant species among NCC, CC10, and CC30 soil samples.

Supplementary Figure 2 Dynamic changes in differentially regulated genera abundance of bacteria **(A)** and fungi **(B)**.

Supplementary Table 1 Overview of the assembled sugarcane soil microorganism metagenome.

Supplementary Table 2 Analysis of similarities analysis results at the genus level for the bacterial and fungal communities and for the gene families of the KEGG database and the CAZy database.

Supplementary Table 3.1 Differential analysis of CC10 and NCC in bacterial community.

Supplementary Table 3.2 Differential analysis of CC30 and NCC in bacterial community.

Supplementary Table 3.3 Differential analysis of CC30 and CC10 in bacterial community.

Supplementary Table 4.1 Differential analysis of CC10 and NCC in fungal community.

Supplementary Table 4.2 Differential analysis of CC30 and NCC in fungal community.

Supplementary Table 4.3 Differential analysis of CC30 and CC10 in fungal community.

Supplementary Table 5.1 KEGG pathway classification.

Supplementary Table 5.2 KEGG pathway abundance.

Supplementary Table 5.3 KEGG level abundance.

Supplementary Table 5.4 KEGG module abundance.

Supplementary Table 5.5 KEGG KO abundance.

Supplementary Table 5.6 KEGG enzyme abundance.

Supplementary Table 6.1 Carbohydrate-active enzyme function annotation abundance in class level.

Supplementary Table 6.2 Carbohydrate-active enzyme function annotation abundance in family level.

Supplementary Table 7 The VIF value of the environment factor.

Supplementary Table 8 Spearman’s analysis between the environmental factors.

Supplementary Table 9.1 Spearman’s correlation between KEGG pathways and environmental factors of bacterial community.

Supplementary Table 9.2 Spearman’s correlation between KEGG pathways and environmental factors of fungal community.

Supplementary Table 10 Descriptions and abbreviations of nitrogen and sulfur metabolism functional genes.

Supplementary Table 11 Difference significance analysis based on LSD test between different groups.
