# Supplementary figures and images for: Soil Metagenomics Reveals Effects of Continuous Sugarcane Cropping on the Structure and Functional Pathway of Rhizospheric Microbial Community
Source: Front Microbiol. 2021 Mar 5;12:627569. doi: 10.3389/fmicb.2021.627569 (PMC7973049; doi:10.3389/fmicb.2021.627569)

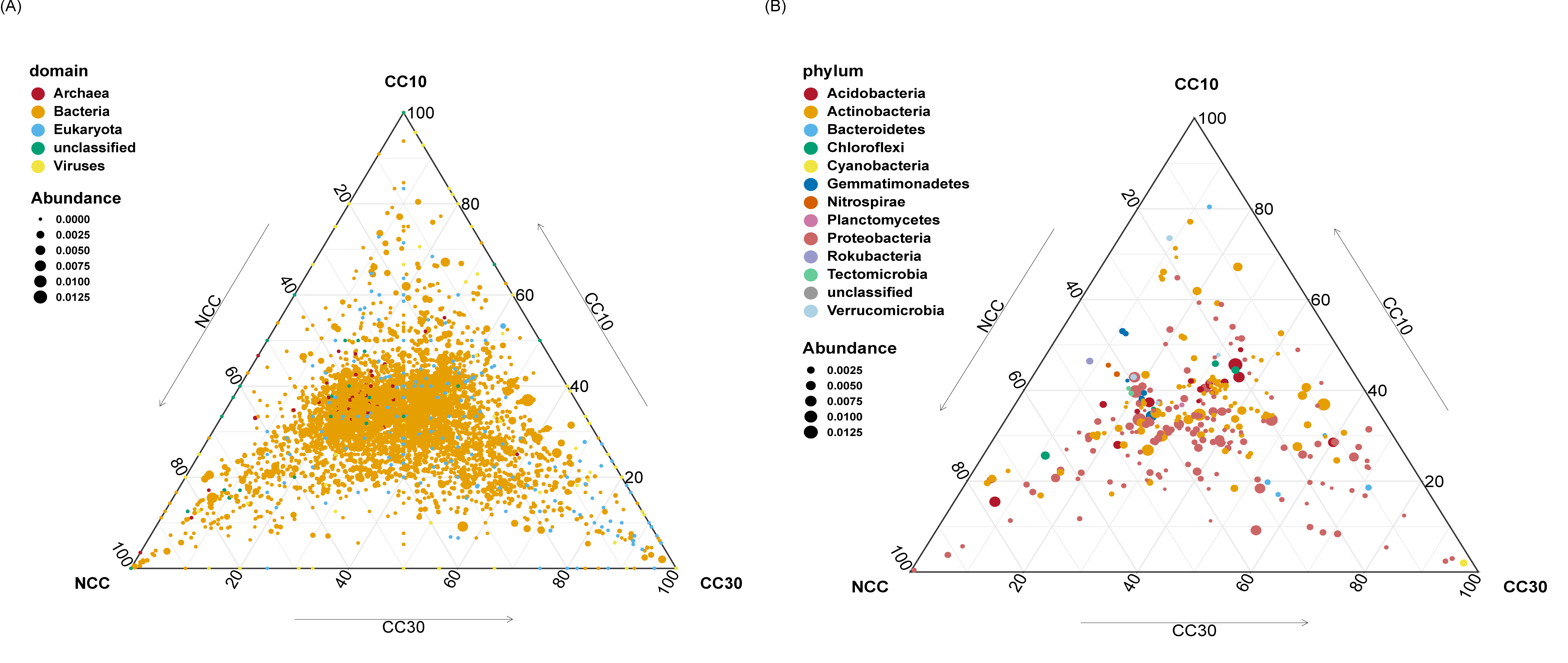

Supplement: Supplementary Figure 1 — Ternary plot depicting RAs of soil microorganisms in three sugarcane soil samples. (A) Ternary plot of domain level from all species among NCC, CC10, and CC30 soil samples. (B) Ternary plot of phylum level from top 300 most abundant species among NCC, CC10, and CC30 soil samples. [file Image_1.TIF]

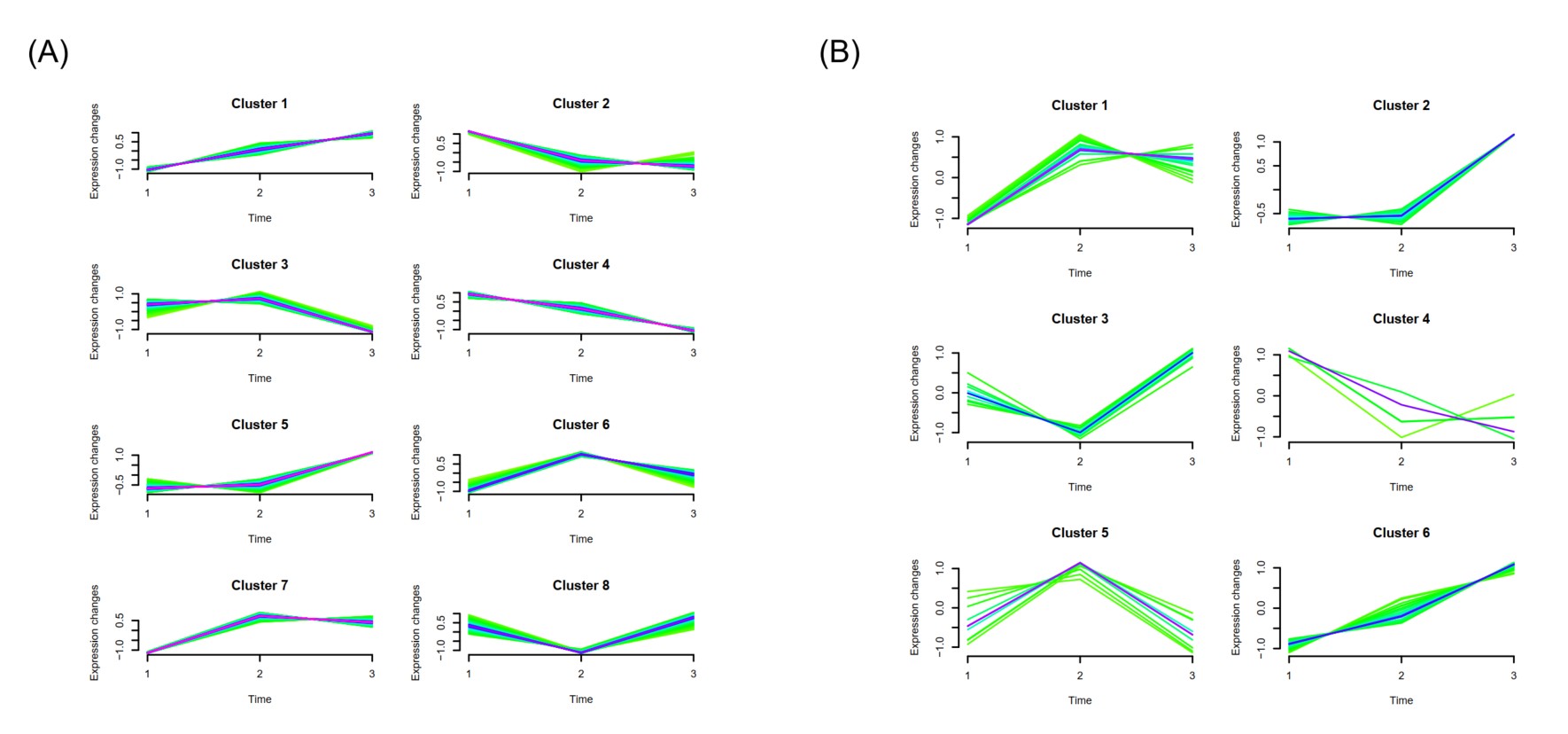

Supplement: Supplementary Figure 2 — Dynamic changes in differentially regulated genera abundance of bacteria (A) and fungi (B). [file Image_2.JPEG]
